# Cytogenetic alterations in ovarian clear cell carcinoma detected by comparative genomic hybridisation

**DOI:** 10.1038/sj.bjc.6600896

**Published:** 2003-05-13

**Authors:** J Dent, G D Hall, N Wilkinson, T J Perren, I Richmond, A F Markham, H Murphy, S M Bell

**Affiliations:** 1 Cancer Research UK Clinical Cancer Centre in Leeds, St James's University Hospital, Leeds LS9 7TF, UK; 2 Hull Royal Infirmary, Hull HU3 2JZ, UK; 3 Molecular Medicine Unit, University of Leeds, St James's University Hospital, Leeds LS9 7TF, UK; 4 Academic Unit of Pathology, University of Leeds, Leeds LS1 3EX, UK

**Keywords:** clear cell ovarian, CGH, LOH, chromosome 9

## Abstract

Ovarian clear cell carcinoma (OCCC) accounts for a small but significant proportion of all ovarian cancers and is a distinct clinical and pathological entity. It tends to be associated with poorer response rates to chemotherapy and with a worse prognosis. Little is known about possible underlying genetic changes. DNA extracted from paraffin-embedded samples of 18 pure OCCC cases was analysed for genetic imbalances using comparative genomic hybridisation (CGH). All of the 18 cases showed genomic alterations. The mean number of alterations detected by CGH was 6 (range 1–15) indicating a moderate level of genetic instability. Chromosome deletions were more common than amplifications. The most prominent change involved chromosome 9 deletions in 10 cases (55%). This correlates with changes seen in other epithelial ovarian cancers. This deletion was confirmed using microsatellite markers to assess loss of heterozygosity (LOH) at four separate loci on chromosome 9. The most distinct region of loss detected was around the *IFNA* marker at 9p21 with 41% (11 out of 27cases) LOH. Other frequent deletions involved 1p (five out of 18; 28%); 11q (four out of 18; 22%) and 16 (five out of 18; 28%). Amplification was most common at chromosome 3 (six out of 18; 33%); 13q (four out of 18; 22%) and 15 (three out of 18; 17%). No high-level amplifications were identified. These features may serve as useful prognostic indicators in the management of OCCC.

Ovarian malignancy is the fourth leading cause of death from cancer in women in the UK, and demonstrates an overall 5-year survival rate of 33% (Gatta *et al*, 1998). The common histological subtypes of epithelial ovarian cancer are serous, mucinous and endometrioid, which together account for more approximately 85% of cases (Clark *et al*, 2001). Clear cell ovarian carcinoma is a less common subtype of ovarian cancer and accounts for 5–10% of all ovarian malignancies (Kennedy *et al*, 1989). Although morphologically distinct, ovarian clear cell carcinoma (OCCC) remains controversial in terms of pathological characteristics and grading, response to treatment and overall prognosis.

The identification of clear cell histology has been shown to be an indicator of poor prognosis in many studies (Sugiyama *et al*, 2000; Clark *et al*, 2001), with an inferior response to platinum-based chemotherapy (Goff *et al*, 1996) and an increased incidence of cancer-related complications, such as thromboembolic disease (Recio *et al*, 1996) and malignancy-related hypercalcaemia (Koshiyama *et al*, 1999). Although other studies have contested this view (O'Brien *et al*, 1993), many clinicians consider clear cell histology as an indication for chemotherapy in patients with otherwise low-risk disease (FIGO stage Ia/Ib).

Cytogenetic and molecular analysis of ovarian cancers has detected a number of structural cytogenetic abnormalities. Published karyotypic analyses have tended to include epithelial ovarian cancers of all histological subtypes and have shown frequent abnormalities of chromosomes 1, 3, 6, 11, 17 and 19, with less frequent abnormalities of chromosomes 2, 4, 5, 9 and 21 (Gallion *et al*, 1990; Taetle *et al*, 1999; Roberts and Tattersall, 1990). The development of improved molecular techniques including the use of polymorphic genetic markers has provided more precise ways to identify these genetic abnormalities.

Comparative genomic hybridisation (CGH) is an analytical technique using a single hybridisation with an equal mixture of distinct fluorescently labelled normal and tumour DNA that permits identification of regions of chromosomes that have undergone either an increase or a decrease in DNA amount, causing genomic imbalance in the tumour. Thus, it can be used to examine the entire genome for amplifications and deletions using extracted tumour DNA (Kallioniemi *et al*, 1992; Visakorpi, 1995).

Comparative genomic hybridisation analysis has revealed numerous cytogenetic changes in epithelial ovarian cancers (Sonoda *et al*, 1997) with common sites of amplification, in order of frequency, at 8q, 20q, 3q, 1q, 20p, 9p and 12p along with deletions at 5q, 9q, 17p, 4q, 16q and 22q. Comparative genomic hybridisation has detected aberrations that correlate with ovarian tumour grade including deletions at chromosome 11p and 13q, and amplifications at 8q and 7p in poorly differentiated tumours as compared with 12p deletions and 18p amplifications in well- and moderately differentiated tumours (Kiechle *et al*, 2001). Some evidence suggests that the main different histological subtypes (serous, mucinous and endometrioid) have different copy number karyotypes, with gains more frequently noted at 10q and 11q in endometrioid and serous tumours respectively and at 17q in mucinous tumours (Tapper *et al*, 1997).

Previously published cytogenetic analyses of ovarian cancer have included very few cases with clear cell histology. The aim of this study was therefore to characterise the genetic alterations of a cohort of pure clear cell ovarian tumours.

## MATERIALS AND METHODS

### Tumour samples

Cases of pure clear cell carcinoma of the ovary were identified from the pathological archives of three specialist cancer hospitals (St James's University Hospital, Leeds General Infirmary and Hull Royal Infirmary). All cases were reviewed and clear cell morphology confirmed independently by two expert gynaecological pathologists (NW and IR). Demographic and survival data were collected anonymously.

### DNA extraction

DNA was extracted from paraffin-embedded samples using standard techniques (Jackson *et al*, 1990). Briefly, 10 *μ*m tumour sections were cut from paraffin wax samples. Sections were de-waxed in xylene and rehydrated through graded alcohol. Relevant areas of individual sections were selected manually, dissected and then digested for 5 days at 37°C with proteinase K (0.1 mg ml^−1^, Sigma, UK) followed by extraction twice with phenol : chloroform : isoamyl alcohol and once with chloroform : isoamyl alcohol. The DNA was then precipitated with ethanol, collected by centrifugation, air-dried and resuspended in sterile distilled water.

### Comparative genomic hybridisation

Comparative genomic hybridisation was performed using fluoro-chrome-conjugated DNAs (Kallioniemi *et al*, 1992; Visakorpi *et al*, 1995). In brief, tumour DNA was labelled with Spectrum Green (Vysis, UK) and normal human genomic placental reference DNA with Spectrum Red (Vysis, UK) by nick translation. Normal human lymphocyte metaphase preparations (Vysis, UK) were denatured at 72–74°C for 5 min in a denaturation solution (70% formamide, 2 × SSC, pH 5.3), and dehydrated in an ethanol series (70, 85, then 100%). A probe mixture comprising 400ng of labelled tumour DNA, 400ng of labelled reference DNA and 20 *μ*g of Cot-1 DNA (Gibco BRL, UK) was denatured at 72–74°C for 5 min and applied to the normal lymphocyte metaphases and cohybridised at 37°C for 2 days. Posthybridisation washes were carried out in 0.4 × SSC/0.3% (v v^−1^) NP-40 at 72–74°C for 2 min, followed by 2 × SSC/0.1% (v v^−1^) NP-40 for 2 min at 20°C. Slides were air-dried and counterstained with 4,6-diamidino-2-phenylindole (DAPI) in Vectashield antifade solution.

### Digital image analysis

Hybridised metaphase spread images were visualised using a Zeiss Axioplan fluorescence microscope. Metaphase images for the three different colours blue (DAPI), Spectrum Red and Spectrum Green were collected using a cooled CCD camera (SenSys 1400) and stored as a three-colour image using the Vysis Quantitative Image Processing System (QUIPS). The chromosomes were identified based on their DAPI banding. The green-to-red fluorescence ratio profiles were automatically determined for each chromosome and a mean ratio profile combining 5–10 metaphase spreads was generated. A ratio of below 0.85 or above 1.15 was taken to indicate regions of under-representation (losses) or over-representation (gains), respectively. If the red–green ratio exceeded 1.5 in a small segment of a chromosome arm, the regions were considered to represent a high level of DNA amplification.

### Loss of heterozygosity

Loss of heterozygosity (LOH) in a total of 27 tumour specimens was examined with the following microsatellite markers: *IFNA* (9p21); D9S104 (9p13); *HXB* (9q32–34); D9S64 (9q34). The PCR reaction mixture contained 12.5 pmol of each primer, one of which was fluorescently labelled, 0.75 U *Taq* DNA polymerase (Promega, UK), 1.5 mM MgCl_2_, 200 *μ*M each of dATP, dCTP, dTTP, dGTP and 50 ng of sample DNA in a 20 *μ*l reaction volume. PCR amplification was performed in a thermal-cycler (Perkin-Elmer, UK). The conditions for amplification were 95°C for 5 min, then 40 cycles of 95° for 30 s, 58° for 45 s, and 72°C for 45 s with a final extension at 72°C for 5 min.The PCR products were denatured and run on a 6% polyacrylamide denaturing gel in 1 × Tris Boric Acid (TBE) buffer on a Model 377 Applied Biosystems automated fluorescent DNA sequencer, using a four-colour detection system. One microlitre from each PCR was combined with 4 *μ*l formamide and 0.5 *μ*l of a fluorescent size marker solution (Applied Biosystems, UK). This mixture was denatured for 3 min at 90°C after which 5 *μ*l was loaded into each well on the prewarmed gel. The gel was run for 4 h at 30 W and 50°C.

The fluorescent gel data collected during the run were analysed using the Genescan Analysis program (ABI) at the end of the run. Allelic imbalance indicative of LOH was scored when there was more than 50% loss of intensity of one allele in the tumour sample compared to the matched allele from normal tissue (Cawkwell *et al*, 1993).

## RESULTS

Thirty three cases of OCCC, presenting between 1976 and 1999, were identified from the pathological records of three institutions. Comparative genomic hybridisation was performed on 18 of these cases (case numbers 1–18). Twelve of the 18 CGH cases had sufficient tumour and normal material for confirmatory LOH markers also to be analysed. Loss of hetrozygosity marker analysis was also performed on an additional 15 cases (case numbers 19–33) for which paired tumour and normal samples could be identified. In total, LOH was performed on 27 of the 33 OCCC cases.

### Clinical results

The median age at diagnosis was 57 years (range 37–84). There were 20 (61%) FIGO stage I, one (3%) FIGO stage II, nine (27%) FIGO stage III and three (9%) FIGO stage IV cases. The median actuarial overall survival for all 33 cases was 3.7 years with a 5 year overall survival rate of 45% (95% CI 28–62%).

### CGH results

**Figure 1:**
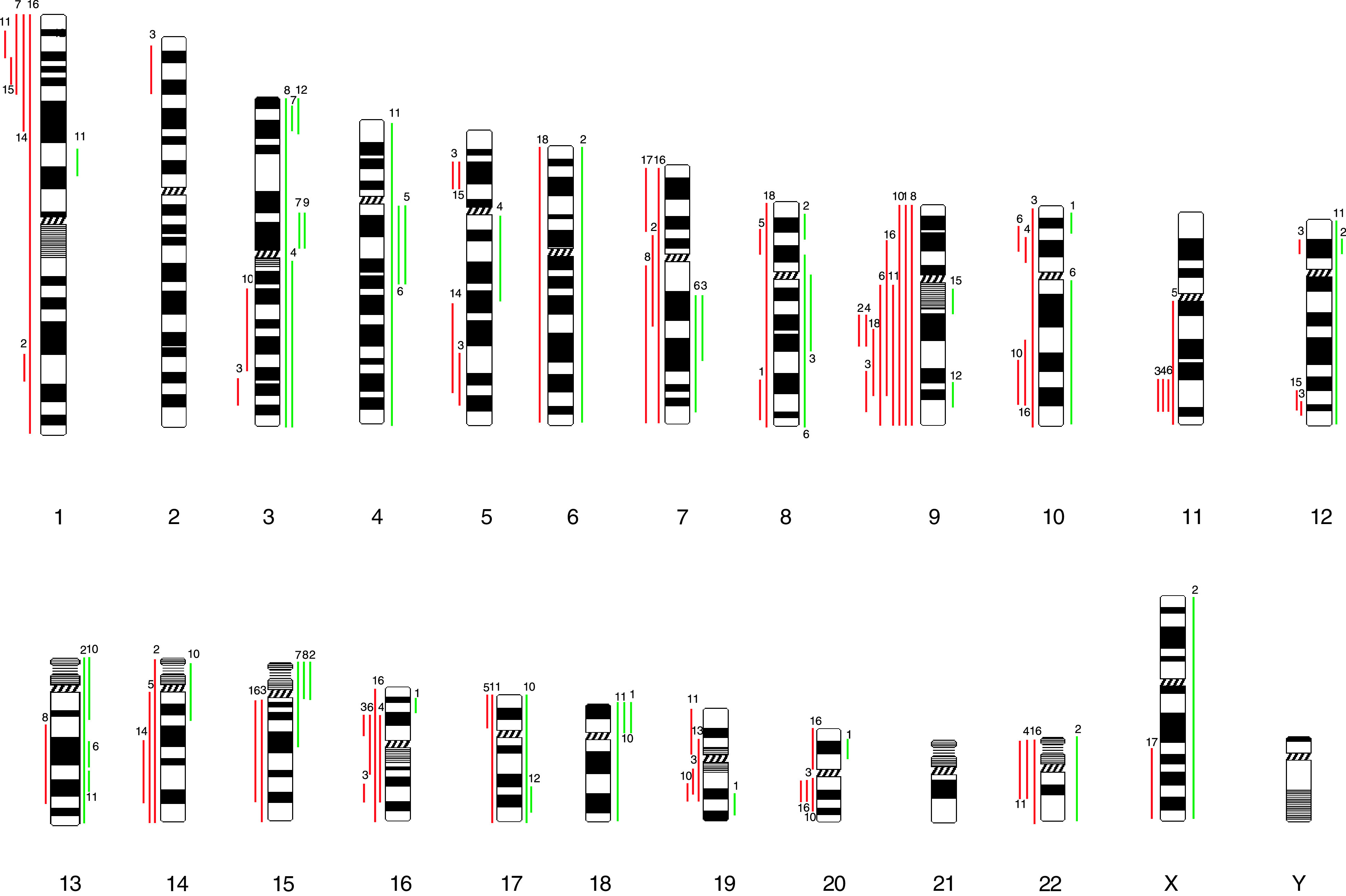
Schematic representation of the chromosomal imbalances detected by CGH in 18 cases. Red vertical lines on the left-hand side represent chromosome deletion. Green vertical lines on the right-hand side represent chromosome amplification. Each line represents genetic aberrations in one case.

Genomic imbalances were detected in all 18 cases of OCCC examined by CGH (Figure 1

, Table 1

**Table 1:** Comparative genomic hybridisation findings in 18 clear cell ovarian carcinoma cases

**Case**	**Stage**	**CGH results**
1	3c	+10p14, +16p13, +18p, +19q13.3, +20p12 −8q23–24.3, −9
2	1c	+6, +8p22, +12p12, +13, +15p, +22, +X −1q32, −7p13−q22, −9q21, −14
3	1c	+7q21–31, +8cter−p21.3 −2p22–24, −3q26.3, −5p14, −5q31–34, −9q31–34, −10, −11q23, −12p12, −12q24.2, −15q, −16p12, −16q22, −19q13.1, −20q12
4	1c	+3q, +5q11.2–21 –9q21, −10p12, −11q23, −16p12−q22, −22pter–q12
5	3b	+4q12–24 −8p21, −11q, −14q, −17p
6	3b	+4q12–24, +7q21–36, +8p12–qter, +10q, +13q21, −9q, −10p13, −11q23, −16p12–q13
7	1a	+3p25, +3p12, +15pter–q21 −1pter–32
8	1c	+3, +15p −9, −7q, −13q14–32
9	3c	+3p12
10	1a	+13p13–q14, +14p13–q13, +17, +18p −3q13.3–26, −9, −10q23–25, −19q13.2, −20q11.2–13.1
11	1a	+1p22, +4, +12, +13q28, +18 −1p36.1, −9q, −17, −19p, −22pter–q12
12	1c	+3p26–24, +9q33, +17q24
13	1a	−19p13.1–q13.2
14	1c	−1p31–pter, −5q21–33, −14q21–31
15	2a	+9q12 −1p33–35, −5p14, −12q24.1
16	4	−1, −7, −9p21–q33, −10q22–25, −15cter–q25, −16, −20p, −20q12, −22
17	3c	−7p14–pter, −Xq24–qter
18	3c	−6, −8, −9q21–33

). Chromosomal losses were found more frequently than chromosomal gains, with a mean number of CGH changes (gains and losses) per case of 6 (range 1–15). No high-level amplifications were identified at the 1.5 threshold. Chromosome 9 was the most common site for genetic abnormalities with 10 of the 18 (55%) cases showing deletion of 9p and/or 9q frequently altered, again showing a higher prevalence of losses than gains. Other common deletions included chromosome 1p (five cases, 28%), chromosome 11q (four cases, 22%) and chromosome 16p/q (five cases, 28%). The most common sites of amplification were on chromosome 3 (six cases, 33%) and chromosome 13q (four cases, 22%).

### LOH results

**Figure 2:**
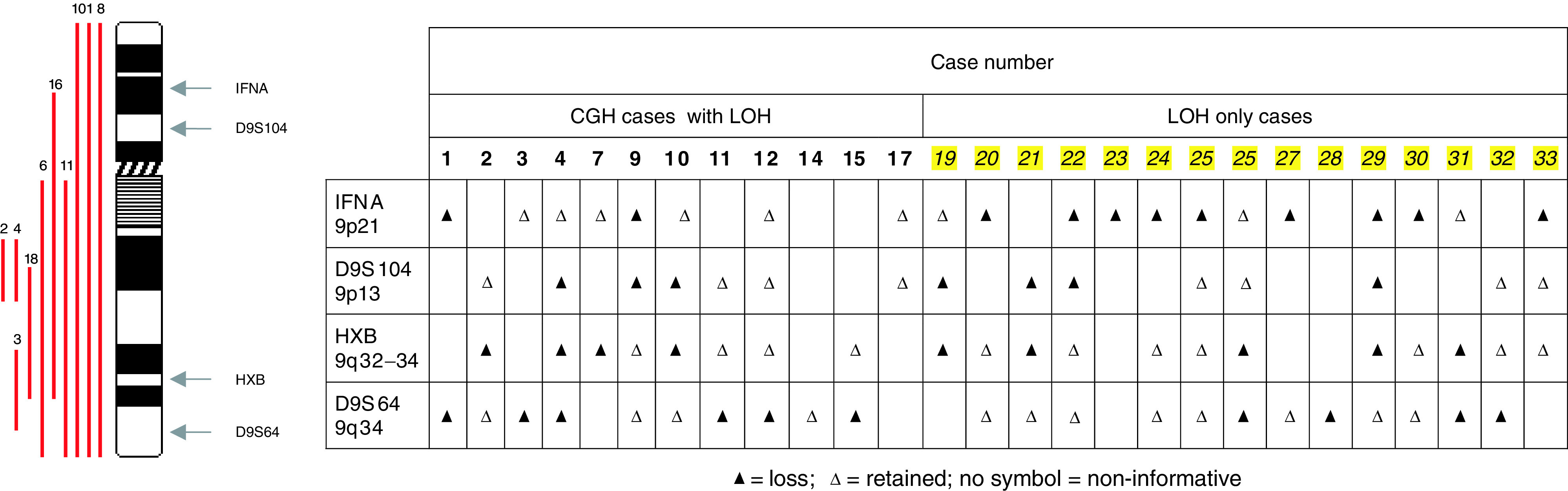
Chromosome 9 LOH marker results with chromosome 9 ideogram. Loss of heterozygosity was performed on a total of 27 cases. The case numbers on the left represent LOH cases for which CGH was also performed (see Table 1). The remaining cases (numbered 1–15) had LOH alone performed.

In view of the high rate of loss on chromosome 9, LOH marker analysis was performed as described. Six cases (case numbers 1, 2, 3, 4, 10 and 11) that had loss seen on chromosome 9 with CGH had confirmatory LOH studies carried out. Loss of heterozygosity was identified in at least one locus on chromosome 9 in all of these six cases. In total, combining all 27 LOH cases, LOH was observed in 11 cases (41%) at *IFNA* marker (9p21), seven cases (26%) at D9S104 (9p13), nine cases (33%) at *HXB* (9q32–34) and 10 cases (37%) at D9S64 (9q34) (Figure 2

).

## DISCUSSION

This study has assessed the cytogenetic abnormalities in a cohort of ovarian cancers with pure clear cell histology. Comparative genomic hybridisation performed on 18 cases identified frequent deletions of chromosome 9p, 1p, 11q and 16p/q and amplifications of 3 and13q.

Analysis of the three main histological ovarian subtypes (serous, mucinous and endometrioid) has demonstrated distinct karyotypes with gains at 17q more frequent in mucinous tumours with gains at 1q, 10q and 11q seen more commonly in serous and endometrioid tumours (Tapper *et al*, 1997). However, CGH analysis of fallopian tube carcinoma has demonstrated similarities with serous carcinoma of both uterus and ovary (Pere *et al*, 1998). Our results suggest that the pattern of genomic alterations found in clear cell ovarian tumours is distinct from that seen in other ovarian histological subtypes. A recent analysis of ovarian cancer using oligonucleotide microarrays has demonstrated distinct patterns of gene expression for the different morphological subtypes. In clear cell tumours, 73 genes, with a two- to 29-fold increase in expression, were identified which distinguished it from other ovarian subtypes (Schwartz *et al*, 2002).

The cytogenetics of 12 cases of clear cell ovarian carcinoma assessed by CGH has been reported by Suehiro *et al* (2000). This Japanese study, which included eight cases of FIGO stage I disease, demonstrated amplification of chromosome 8q in eight cases (67%). Increased copy number was also frequently identified on chromosomes 17q and 20q; reduced copy number of 19p was also identified; chromosome 9 deletions were only seen in two cases (17%). No LOH analysis was performed either to confirm or further define these changes.

These results are distinct to those obtained in the current study, where amplification of chromosome 8 was infrequent and deletion of chromosome 9p and q was common. The reason for these differences is not clear. However, in many Japanese studies the incidence of clear cell histology in epithelial ovarian carcinoma is as high as 15.3% (Sugiyama *et al*, 2000). This differs significantly from an incidence of approximately 5% seen in American or European studies (Omura *et al*, 1991; O'Brien *et al*, 1993). It is not clear whether this reflects differences in the diagnostic criteria used to define the clear cell phenotype in Japan, or differences in the molecular genetics of ovarian cancer in Japanese women. Other CGH studies of ovarian cancer of all histological subtypes have identified deletions of chromosome 9p; a study of 24 cases of grade 1 or 2 ovarian cancers reported 9p loss in six cases (25%; (Tapper *et al*, 1997)). A larger study of 106 cases of all histological grades identified 9p loss in 41% of patients (Kiechle *et al*, 2001).

The LOH analysis of chromosome 9 performed herein, confirmed the CGH results obtained in this study and was consistent with other published data (Chenevix-Trench *et al*, 1994). A detailed analysis of chromosome 9 in 33 ovarian tumours of various grades and stages detected LOH in 26 cases (79%) and proposed three specific regions of deletion – one at 9p21 and two on 9q; one proximal, one distal (Devlin *et al*, 1996). During the last few years, these three regions of deletion have been examined in detail in attempts to identify tumour suppressor genes whose function is lost in a variety of ovarian cancers.

The locus at 9p21, deleted in 11 of the 20 informative cases (55%) in this study, includes the cyclin-dependent kinase inhibitor 2 (*CDKN2*) locus, a region now known to encode the proteins *CDKN2A*/p16(*INK4A*), *CDKN2B*/p15(*INK4B*) and an alternative reading frame of *CDKN2A*, p14ARF (Kamb *et al*, 1994; Stott *et al*, 1998). These three proteins act as tumour suppressor genes through the two central growth control pathways, which act upon Rb and p53 (Saegusa *et al*, 2001). Deletion or inactivation of genes within this region is a frequent cytogenetic abnormality in other malignancies such as melanoma (Kamb *et al*, 1994), bladder cancer (Cairns *et al*, 1995) and renal cancer (Schraml *et al*, 2001). In the past, OCCC has been confused with metastatic renal cell carcinoma (Young and Hart, 1992) and it is interesting to note that chromosome 9 loss has also been found in renal clear cell tumour with 33% having partial or complete deletion of chromosome 9 (Cairns *et al*, 1995).

The data we have obtained suggest a role for one or more of the gene products from this locus in the molecular pathogenesis of clear cell ovarian carcinoma. The expression of *CDKN2A*/p16(*INK4A*) in ovarian carcinoma has been assessed in a number of studies with conflicting results. P16 overexpression has been reported in 22 of 24 (90%) high-stage malignant ovarian tumours (Shigemasa *et al*, 1997), whereas other reports suggest a correlation between p16 underexpression and low-grade ovarian tumours; 22 out of 60 (37%) (Fujita *et al*, 1997).

The deletions seen within chromosome 9q by both CGH and LOH in our study may also imply a role for alternative tumour suppressor genes in the molecular pathogenesis of clear cell ovarian carcinoma. The distal locus on 9q includes two genes with potential roles as tumour suppressor genes in ovarian cancer; the *TSC1* gene at 9q34 (Miloloza *et al*, 2000) and the *DBCCR1* gene at 9q32–33 (Habuchi *et al*, 1998). The specific roles of these genes in the pathogenesis and prognosis of ovarian cancer have not previously been assessed.

The other frequent genetic abnormalities seen in this study include deletion on chromosome 16p and 16q and amplification of chromosome 3p and 3q. Allele loss on 16q has been connected with progression of some cancer types, including breast and lung (Lee, 1996; Sato *et al*, 1998). 16q deletions were observed in 14 of 21 (67%) high-grade epithelial ovarian tumours (Kawakami *et al*, 1999). Candidate genes that map to this region include *CDH13* (H-cadherin; 16q24). This gene belongs to the family of cell adhesion molecules of which E-cadherin is a well-recognised tumour suppressor gene in gastric cancer (Becker *et al*, 1994). Copy number increases of chromosome 3q in ovarian carcinomas have previously been reported (Arnold *et al*, 1996; Sonoda *et al*, 1997) and mapped to a region at 3q26 containing *PIK3CA* (Shayesteh *et al*, 1999). Furthermore, upregulation of this gene has been seen in 36% of malignant, mostly serous, high-grade ovarian carcinomas (Sonoda *et al*, 1997). *PIK3CA* encodes a subunit of a phosphatidylinositol 3-kinase involved in kinase-mediated cell signalling (Rodriguez-Viciana *et al*, 1996). Other published work has also found that copy number alterations in ovarian cancer correlates to histological tumour grade with an overall increase in DNA sequence copy number abnormalities seen in high-grade tumours as opposed to low-grade tumours (Iwabuchi *et al*, 1995). Specifically, 3q alterations were reported in high-grade tumour cases with an increased copy number on 3q25–26 found in 13 out of 26 cases (50%).

The cytogenetic abnormalities detected by the present study reflect many of those previously identified in other histological subtypes of ovarian cancer (Sonoda *et al*, 1997; Tapper *et al*, 1997). No genetic change unique to the clear cell histology was identified. Further characterisation of candidate genes within the regions of deletion/amplification is underway.

## References

[bib1] Arnold N, Hagele L, Walz L, Schempp W, Pfisterer J, Bauknecht T, Kiechle M (1996) Overrepresentation of 3q and 8q material and loss of 18q material are recurrent findings in advanced human ovarian cancer. Genes Chromosomes Cancer 16: 46–54 9162197 10.1002/(SICI)1098-2264(199605)16:1<46::AID-GCC7>3.0.CO;2-3

[bib2] Becker KF, Atkinson MJ, Reich U, Becker I, Nekarda H, Siewert JR, Hofler H (1994) E-cadherin gene mutations provide clues to diffuse type gastric carcinomas. Cancer Res 54: 3845–3852 8033105

[bib3] Cairns P, Tokino K, Eby Y, Sidransky D (1995) Localization of tumour suppressor loci on chromosome 9 in primary human renal cell carcinomas. Cancer Res 55: 224–227 7812948

[bib4] Cawkwell L, Bell SM, Lewis FA, Dixon MF, Taylor GR, Quirke P (1993) Rapid detection of allele loss in colorectal tumours using microsatellites and fluorescent DNA technology. Br J Cancer 67: 1262–1267 8512811 10.1038/bjc.1993.236 PMC1968523

[bib5] Chenevix-Trench G, Kerr J, Friedlander M, Hurst T, Sanderson B, , Coglan M, Ward B, Leary J, Khoo SK (1994) Homozygous deletions on the short arm of chromosome 9 in ovarian adenocarcinoma cell lines and loss of heterozygosity in sporadic tumours. Am J Hum Genet 55: 143–149 8023842 PMC1918224

[bib7] Clark TG, Stewart ME, Altman DG, Gabra H, Smyth JF (2001) A prognostic model for ovarian cancer. Br J Cancer 85: 944–952 11592763 10.1054/bjoc.2001.2030 PMC2375096

[bib8] Devlin J, Elder PA, Gabra H, Steel CM, Knowles MA (1996) High frequency of chromosome 9 deletion in ovarian cancer: evidence for three tumour-suppressor loci. Br J Cancer 73: 420–423 8595153 10.1038/bjc.1996.75 PMC2074458

[bib9] Fujita M, Enomoto T, Haba T, Nakashima R, Sasaki M, Yoshino K, Wada H, Buzard GS, Matsuzaki N, Wakasa K, Murata Y (1997) Alteration of p16 and p15 genes in common epithelial ovarian tumours. Int J Cancer 74: 148–155 9133447 10.1002/(sici)1097-0215(19970422)74:2<148::aid-ijc2>3.0.co;2-z

[bib10] Gallion HH, Powell DE, Smith LW, Morrow JK, Martin AW, van Nagell JR, Donaldson ES (1990) Chromosome abnormalities in human epithelial ovarian malignancies. Gynecol Oncol 38: 473–477 2227564 10.1016/0090-8258(90)90094-2

[bib11] Gatta G, Lasota MB, Verdecchia A (1998) Survival of European women with gynaecological tumours, during the period 1978–1989. EUROCARE Working Group. Eur J Cancer 34: 2218–2225 10070290 10.1016/s0959-8049(98)00326-8

[bib12] Goff BA, Sainz dlC, Muntz HG, Fleischhacker D, Ek M, Rice LW, Nikrui N, Tamimi HK, Cain JM, Greer BE, Fuller AF (1996) Clear cell carcinoma of the ovary: a distinct histologic type with poor prognosis and resistance to platinum-based chemotherapy in stage III disease. Gynecol Oncol 60: 412–417 8774649 10.1006/gyno.1996.0065

[bib13] Habuchi T, Luscombe M, Elder PA, Knowles MA (1998) Structure and methylation-based silencing of a gene (DBCCR1) within a candidate bladder cancer tumor suppressor region at 932–933. Genomics 48: 277–288 9545632 10.1006/geno.1997.5165

[bib14] Iwabuchi H, Sakamoto M, Sakunaga H, Ma YY, Carcangiu ML, Pinkel D, Yang-Feng TL, Gray JW (1995) Genetic analysis of benign, low-grade, and high-grade ovarian tumors. Cancer Res 55: 6172–6180 8521410

[bib15] Jackson DP, Lewis FA, Taylor GR, Boylston AW, Quirke P (1990) Tissue extraction of DNA and RNA and analysis by the polymerase chain reaction. J Clin Pathol 43: 499–504 1696290 10.1136/jcp.43.6.499 PMC502506

[bib17] Kallioniemi A, Kallioniemi OP, Sudar D, Rutovitz D, Gray JW, Waldman F, Pinkel D (1992) Comparative genomic hybridization for molecular cytogenetic analysis of solid tumors. Science 258: 818–821 1359641 10.1126/science.1359641

[bib18] Kamb A, Gruis NA, Weaver-Feldhaus J, Liu Q, Harshman K, Tavtigian SV, Stockert E, Day III RS, Johnson BE, Skolnick MH (1994) A cell cycle regulator potentially involved in genesis of many tumor types. Science 264: 436–440 8153634 10.1126/science.8153634

[bib19] Kawakami M, Staub J, Cliby W, Hartmann L, Smith DI, Shridhar V (1999) Involvement of H-cadherin (CDH13) on 16q in the region of frequent deletion in ovarian cancer. Int J Oncol 15: 715–720 10493953 10.3892/ijo.15.4.715

[bib20] Kennedy AW, Biscotti CV, Hart WR, Webster KD (1989) Ovarian clear cell adenocarcinoma. Gynecol Oncol 32: 342–349 2920955 10.1016/0090-8258(89)90637-9

[bib21] Kiechle M, Jacobsen A, Schwarz-Boeger U, Hedderich J, Pfisterer J, Arnold N (2001) Comparative genomic hybridization detects genetic imbalances in primary ovarian carcinomas as correlated with grade of differentiation. Cancer 91: 534–540 11169935

[bib22] Koshiyama M, Fujii H, Konishi M, Nanno H, Hayashi M, Tauchi K, Yoshida M (1999) Recurrent clear cell carcinoma of the ovary changing into producing parathyroid hormone-related protein (PTH-rP) with hypercalcemia. Eur J Obstet Gynecol Reprod Biol 82: 227–229 10206422 10.1016/s0301-2115(98)00165-1

[bib24] Lee SW (1996) H-cadherin, a novel cadherin with growth inhibitory functions and diminished expression in human breast cancer. Nat Med 2: 776–782 8673923 10.1038/nm0796-776

[bib25] Miloloza A, Rosner M, Nellist M, Halley D, Bernaschek G, Hengstschlager M (2000) The TSC1 gene product, hamartin, negatively regulates cell proliferation. Hum Mol Genet 9: 1721–1727 10915759 10.1093/hmg/9.12.1721

[bib27] O'Brien ME, Schofield JB, Tan S, Fryatt I, Fisher C, Wiltshaw E (1993) Clear cell epithelial ovarian cancer (mesonephroid): bad prognosis only in early stages. Gynecol Oncol 49: 250–254 8504995 10.1006/gyno.1993.1117

[bib28] Omura GA, Brady MF, Homesley HD, Yordan E, Major FJ, Buchsbaum HJ, Park RC (1991) Long-term follow-up and prognostic factor analysis in advanced ovarian carcinoma: the Gynecologic Oncology Group experience. J Clin Oncol 9: 1138–1150 1904477 10.1200/JCO.1991.9.7.1138

[bib29] Pere H, Tapper J, Seppala M, Butzow R (2002) Genomic alterations in fallopian tube carcinoma: comparison to serous uterine and ovarian carcinoma reveals similarity suggesting likeliness in molecular pathogenesis. Cancer Res 58: 4274–4276 9766651

[bib30] Recio FO, Piver MS, Hempling RE, Driscoll DL (1996) Lack of improved survival plus increase in thromboembolic complications in patients with clear cell carcinoma of the ovary treated with platinum *versus* nonplatinum-based chemotherapy. Cancer 78: 2157–2163 8918409 10.1002/(sici)1097-0142(19961115)78:10<2157::aid-cncr17>3.0.co;2-y

[bib31] Roberts CG, Tattersall MH (2001) Cytogenetic study of solid ovarian tumours. Cancer Genet Cytogenet 48(2): 243–253. 10.1016/0165-4608(90)90127-v 2397455

[bib32] Rodriguez-Viciana P, Marte BM, Warne PH, Downward J (1996) Phosphatidylinositol 3′ kinase: one of the effectors of Ras. Philos Trans R Soc London B Biol Sci 351: 225–231 8650270 10.1098/rstb.1996.0020

[bib33] Sato M, Mori Y, Sakurada A, Fujimura S, Horii A (1998) The H-cadherin (CDH13) gene is inactivated in human lung cancer. Hum Genet 103: 96–101 9737784 10.1007/s004390050790

[bib34] Saegusa M, Machida BD, Okayasu I (2001) Possible associations among expression of p14(ARF), p16(INK4a), p21(WAF/CIP1), p27(KIP1), and p53 accumulation and the balance of apoptosis and cell proliferation in ovarian carcinomas. Cancer 92: 1177–1189 11571731 10.1002/1097-0142(20010901)92:5<1177::aid-cncr1436>3.0.co;2-5

[bib35] Schraml P, Struckmann K, Bednar R, Fu W, Gasser T, Wilber K, Kononen J, Sauter G, Mihatsch MJ, Moch H (2001) CDKNA2A mutation analysis, protein expression, and deletion mapping of chromosome 9p in conventional clear-cell renal carcinomas: evidence for a second tumor suppressor gene proximal to *CDKN2A*. Am J Pathol 158: 593–601 11159196 10.1016/s0002-9440(10)64001-1 PMC1850295

[bib36] Schwartz DR, Kardia SL, Shedden KA, Kuick R, Michailidis G, Taylor JM, Misek DE, Wu R, Zhai Y, Darrah DM, Reed H, Ellenson LH, Giordano TJ, Fearon ER, Hanash SM, Cho KR. (2002). Gene expression in ovarian cancer reflects both morphology and biological behavior, distinguishing clear cell from other poor-prognosis ovarian carcinomas. Cancer Res 62: 4722–4729. 12183431

[bib37] Shayesteh L, Lu Y, Kuo WL, Baldocchi R, Godfrey T, Collins C, Pinkel D, Powell B, Mills GB, Gray JW (1999) PIK3CA is implicated as an oncogene in ovarian cancer. Nat Genet 21: 99–102 9916799 10.1038/5042

[bib39] Shigemasa K, Hu C, West CM, Clarke J, Parham GP, Parmley TH, Lorourian S, Baker W, O'Brien TJ (1997) p16 overexpression: a potential early indicator of transformation in ovarian carcinoma. J Soc Gynecol Invest 4: 95–102 9101469

[bib41] Sonoda G, Palazzo J, Du MS, Godwin AK, Feder M, Yakushiji M, Testa JR (1997) Comparative genomic hybridization detects frequent over-representation of chromosomal material from 3q26, 8q24, and 20q13 in human ovarian carcinomas. Genes Chromosomes Cancer 20: 320–328 9408747

[bib42] Stott FJ, Bates S, James MC, McConnell BB, Starborg M, Brookes S, Palmero I, Ryan K, Hara E, Vousden KH, Peters G (1998) The alternative product from the human CDKN2A locus p14(ARF), participates in a regulatory feedback loop with p53 and MDM2. EMBO J 17: 5001–5014 9724636 10.1093/emboj/17.17.5001 PMC1170828

[bib43] Suehiro Y, Sakamoto M, Umayahara K, Iwabuchi H, Sakamoto H, Tanaka N, Takeshima N, Yamauchi K, Hasumi K, Akiya T, Sakunaga H, Muroya T, Numa F, Kato H, Tenjin Y, Sugishita T (2000) Genetic aberrations detected by comparative genomic hybridization in ovarian clear cell adenocarcinomas. Oncology 59: 50–56 10895067 10.1159/000012137

[bib44] Sugiyama T, Kamura T, Kigawa J, Terakawa N, Kikuchi Y, Kita T, Suzuki M, Sato I, Taguchi K (2000) Clinical characteristics of clear cell carcinoma of the ovary: a distinct histologic type with poor prognosis and resistance to platinum-based chemotherapy. Cancer 88: 2584–2589 10861437

[bib45] Taetle R, Aickin M, Yang JM, Panda L, Emerson J, Roe D, Adair L, Thompson F, Liu Y, Wisner L, Davis JR, Trent J, Alberts DS (1999) Chromosome abnormalities in ovarian adenocarcinoma: I. Nonrandom chromosome abnormalities from 244 cases. Genes Chromosomes Cancer 25: 290–300 10379876

[bib46] Tapper J, Butzow R, Wahlstrom T, Seppala M, Knuutila S (1997) Evidence for divergence of DNA copy number changes in serous, mucinous and endometrioid ovarian carcinomas. Br J Cancer 75: 1782–1787 9192982 10.1038/bjc.1997.304 PMC2223609

[bib47] Visakorpi T, Kallioniemi AH, Syvanen AC, Hyytinen ER, Karhu R, Tammela T, Isola JJ, Kallioniemi OP (1995) Genetic changes in primary and recurrent prostate cancer by comparative genomic hybridization. Cancer Res 55: 342–347 7529134

[bib49] Young RH, Hart WR (1992) Renal cell carcinoma metastatic to the ovary: a report of three cases emphasizing possible confusion with ovarian clear cell adenocarcinoma. Int J Gynecol Pathol 11: 96–104 1582751

